# Comparison of Wound Outcomes Between Short-Stitch and Long-Stitch Fascial Closure Following Emergency Exploratory Laparotomy: A Prospective Comparative Study

**DOI:** 10.7759/cureus.110435

**Published:** 2026-06-08

**Authors:** Akshita Akshita, Indu B Dubey, Nishith S Mandal, Jatin Chavda, Shourya Vijayvargia, Aashutosh M Hiremath, Pramatheshwara S Aradhya, Dhanesh Sipani, Ayush Baldania, Hinduja Raju

**Affiliations:** 1 Department of General Surgery, Vardhman Mahavir Medical College and Safdarjung Hospital, New Delhi, IND; 2 School of Medicine, All India Institute of Medical Sciences, New Delhi, IND; 3 School of Medicine, Mysore Medical College and Research Institute, Mysore, IND

**Keywords:** emergency laparotomy, fascial closure, long-stitch closure, short-stitch closure, surgical site infection, wound dehiscence

## Abstract

Background

Emergency exploratory laparotomy is associated with a high incidence of postoperative wound complications, including surgical site infection (SSI) and surgical wound dehiscence (SWD). The fascial closure technique, particularly the stitch length and suture length-to-wound length (SL:WL) ratio, plays a crucial role in wound healing. While short-stitch (SS) techniques have demonstrated superiority in elective surgeries, evidence in emergency settings remains limited.

Methods

This prospective, observational, comparative study was conducted over 18 months at a tertiary care teaching hospital. Ninety-four patients undergoing emergency midline exploratory laparotomy were allocated into SS (n = 47) and long-stitch (LS; n = 47) groups using simple random allocation. Fascial closure was performed using continuous size 1-0 polydioxanone (PDS) suture. The same surgical team performed all procedures to minimize inter-operator variability. Outcomes assessed included SSI (Southampton grading), SWD (World Union of Wound Healing Societies (WUWHS) grading), time-to-fascial closure, postoperative pain (Visual Analogue Scale (VAS) scores), length of hospital stay, and the incidence of incisional hernia. Statistical analysis was performed using appropriate parametric and non-parametric tests, with p < 0.05 considered significant.

Results

Overall SSI incidence was significantly lower in the SS group compared to the LS group (10.6% (n = 5/47) vs 27.7% (n = 13/47); p = 0.036). Severe SSI grades were more frequent in the LS group but did not reach statistical significance. SWD incidence was also significantly lower with SS closure (12.8% (n = 6/47) vs 36.2% (n = 17/47); p = 0.016). Mean time-to-fascial closure was significantly longer in the SS group than in the LS group (14.8 ± 4.2 min vs 11.4 ± 2.9 min, p < 0.001). Postoperative pain scores and length of hospital stay did not differ significantly between groups.

Conclusion

SS fascial closure following emergency exploratory laparotomy is associated with significantly reduced SSI and wound dehiscence, with only a modest increase in closure time. Adoption of this technique may improve postoperative wound outcomes in emergency abdominal surgery.

## Introduction

Emergency exploratory laparotomy is a commonly performed life-saving surgical procedure and is associated with substantial postoperative morbidity. Surgical site infection (SSI) and surgical wound dehiscence (SWD) are among the most frequent complications following emergency abdominal surgery and significantly contribute to prolonged hospital stay, increased healthcare costs, and the need for reintervention [1,2]. Compared to elective surgery, emergency laparotomy carries a higher risk of wound complications due to factors such as contamination, bowel edema, hemodynamic instability, anemia, and compromised nutritional status [3,4].

The fascial closure technique plays a pivotal role in determining postoperative wound outcomes. Traditionally, midline laparotomy incisions are closed using large tissue bites placed at wider intervals, commonly referred to as the long-stitch (LS) technique. However, experimental and clinical studies have demonstrated that large tissue bites may impair wound perfusion and increase the risk of infection and wound failure [5,6]. The concept of optimizing the suture length-to-wound length (SL:WL) ratio, with a recommended ratio of at least 4:1, was introduced to enhance fascial healing and reduce wound complications [7].

The short-stitch (SS) technique, characterized by small tissue bites placed close to the wound edge at shorter intervals, has gained increasing acceptance due to its favorable biomechanical and physiological properties. Randomized controlled trials and meta-analyses in elective midline laparotomy have demonstrated significantly lower rates of SSI, wound dehiscence, and incisional hernia with SS closure compared to conventional techniques [8-13]. Improved tissue perfusion and more uniform distribution of tension across the incision are believed to underlie these benefits [14,15].

Despite strong evidence supporting SS closure in elective surgery, from the STITCH Trial [3] and the ESTOIH Trial [7], and guideline recommendations favoring its use [11-13], data evaluating its effectiveness in emergency laparotomy remain limited. This prospective comparative study was therefore undertaken to compare wound outcomes between SS and LS fascial closure following emergency exploratory laparotomy. The primary wound outcome was to evaluate the incidence of surgical site infection, whereas secondary outcomes included the evaluation of SWD, time-to-fascial-closure, postoperative pain, length of hospital stay, and the presence of an incisional hernia.

## Materials and methods

Trial design

This prospective, randomized, comparative, single-center study was conducted in the Department of General Surgery, Vardhman Mahavir Medical College and Safdarjung Hospital, New Delhi, over an 18-month period (from January 20, 2024, to July 30, 2025). The Institutional Review Board/Thesis Protocol Review Committee of Vardhman Mahavir Medical College and Safdarjung Hospital, New Delhi, approved the study protocol. Since it was a randomized comparative study, it was registered with the Clinical Trials Registry of India prior to initiation (Registration number: CTRI/2024/06/069381; Reference Number: REF/2024/05/084468). Reporting of the trial adheres to the recommendations of the updated and extended Consolidated Standards of Reporting Trials (CONSORT) Statement.

Sample size calculation

The sample size was calculated using the formula for the comparison of two independent proportions:

$n = \frac{\left[ z_{1-\alpha/2} \sqrt{2p(1-p)} + z_{1-\beta} \sqrt{p_1(1-p_1) + p_2(1-p_2)} \right]^2}{(p_1 - p_2)^2}$

The parameters for this calculation were derived from the study by de Vries et al. [9], who reported the incidence of wound infection as 42% ($p_1 = 0.42$) for the small-bites suture technique and 58% ($p_2 = 0.58$) for the conventional large-bites suture technique. Taking these values as reference values and to detect a comparable difference with a 95% confidence level ($\alpha = 0.05, z_{1-\alpha/2} = 1.96$) and 80% statistical power ($\beta = 0.20, z_{1-\beta} = 0.84$), the initial required sample size was estimated at 152 patients per arm, totaling 304 participants after entering all the values in the above formula.

Since only about 120 patients meeting the inclusion criteria were expected to be available for the study during the data collection period, applying finite population correction, the final sample size required was reduced to 86 ($n= {304/ (1+304/120)} = 86$). Considering a 10% non-response rate, the final sample size was set at 94 patients, with 47 participants allocated to each study group.

Study population and randomization 

Patients aged more than 12 years undergoing emergency midline exploratory laparotomy were included. Patients with pregnancy, previous abdominal surgery, or conditions unsuitable for primary fascial closure were excluded. 

All patients presenting to the surgical emergency were subjected to history-taking and general examination, including pulse rate, blood pressure, and body temperature, and a thorough clinical abdominal examination was performed. Relevant blood and radiological investigations were also done. For those patients who required emergency laparotomy (based on clinical/imaging findings suggestive of peritonitis or requiring urgent surgery for abdominal pathology), written informed consent was obtained, and simple randomization was performed using the coin toss method (heads = LS, tails = SS). The coin toss was performed by an independent coordinator, not a member of the surgical team. To account for potential confounding factors affecting wound outcomes, baseline patient demographics and baseline values of nutrition in the form of serum albumin and serum hemoglobin were meticulously recorded for both groups. Patients were taken up for emergency exploratory laparotomy under general anesthesia after antibiotic prophylaxis and adequate resuscitation. All surgeries were performed by the same surgical team. Blinding was not performed.

Interventions

After an adequate midline laparotomy incision, exploration of the whole peritoneal cavity was done. Intraoperative findings were noted, and surgery was performed as per the requirement. Following management of intra-abdominal pathology, the sheath closure was performed by the surgery team using either the SS or LS technique, depending on the patient's allocated randomized group.

SS Technique

Sheath closure was performed in a continuous fashion by placing the sutures 5 mm from the rectus edge, with an intersuture distance of 5 mm from the wound edge, using size 1-0 PDS suture, a single 150 cm thread, and an HR 40 mm needle.

LS Technique

Sheath closure was performed in a continuous fashion, with sutures placed 1 cm from the rectus edge and an intersuture distance of 1 cm, using size 1-0 PDS suture, a single 150 cm thread, and an HR 40 mm needle.

Time taken to close the sheath was noted from the first needle bite into the fascia until the completion of the last knot tied over the fascia. This time was defined as the time taken for fascial closure.

The length of the wound was measured for each patient just before starting fascial closure, defined as the shortest distance between the two edges of the abdominal wound at the skin level. Antibiotics were administered postoperatively depending on the pathology found at the surgical site. Postoperatively, the patient was closely monitored. Daily antiseptic dressings were done twice for all patients. Basic analgesics were given (one g of intravenous paracetamol 8 hourly), and patients were assessed daily for any signs of SSI and SWD. The presence of SSI was assessed daily on the morning of postoperative days 1, 3, 5, and 7, graded according to the Southampton grading system [16], and the number of patients with a SSI was noted daily for both groups. Likewise, the presence of SWD was assessed daily on the morning of postoperative days 1, 3, 5, and 7 and was graded according to the World Union of Wound Healing Societies (WUWHS)-Sandy grading system [17], and the number of patients with SWD was noted daily for both groups. An SSI event (any grade) or SWD event (any grade) occurring in a new, unique patient at the end of that given postoperative day was counted towards incidence. Furthermore, the total number of SSI or SWD, counted as previously mentioned, was included in the prevalence. In case of SWD or SSI, appropriate interventions were done.

Postoperative pain was assessed daily on the morning of postoperative days 1, 3, 5, and 7 using the Visual Analogue Scale (VAS), and a score was assigned to each patient for that respective postoperative day.

Outcome measures

The primary outcome of the study was to evaluate the incidence of SSIs in both groups, as characterized by the Southampton Wound Grading System [16]. The secondary outcomes assessed were the incidence of SWD (specifically measured using the WUWHS-Sandy SWD grading system [17]), time-to-fascial closure, postoperative pain during the hospital stay (using the VAS scores), the initial length of hospital stay during the index operation, and the presence of an incisional hernia.

Follow-up

Patients were assessed on postoperative days 1, 3, 5, and 7 during the hospital stay for postoperative pain, SSI, and SWD. They were subsequently followed up in the outpatient department for 12 months. At the 12-month mark, the wound was examined clinically for the presence of an incisional hernia by the same surgical team that conducted the index operation.

Statistical analysis

Data were entered into an Apple Numbers spreadsheet (Apple Inc., Cupertino, CA, USA) and analyzed using the IBM SPSS Statistics for Windows, Version 28.0 (Released 2021; IBM Corp., Armonk, NY, USA). Continuous variables were presented as mean ± standard deviation (SD) and compared using an independent-samples t-test. Categorical variables were expressed as frequencies and percentages. Pearson’s chi-square test or Fisher’s exact test was used for comparison of categorical variables as appropriate. A p-value of less than 0.05 was considered statistically significant.

**Figure 1:**
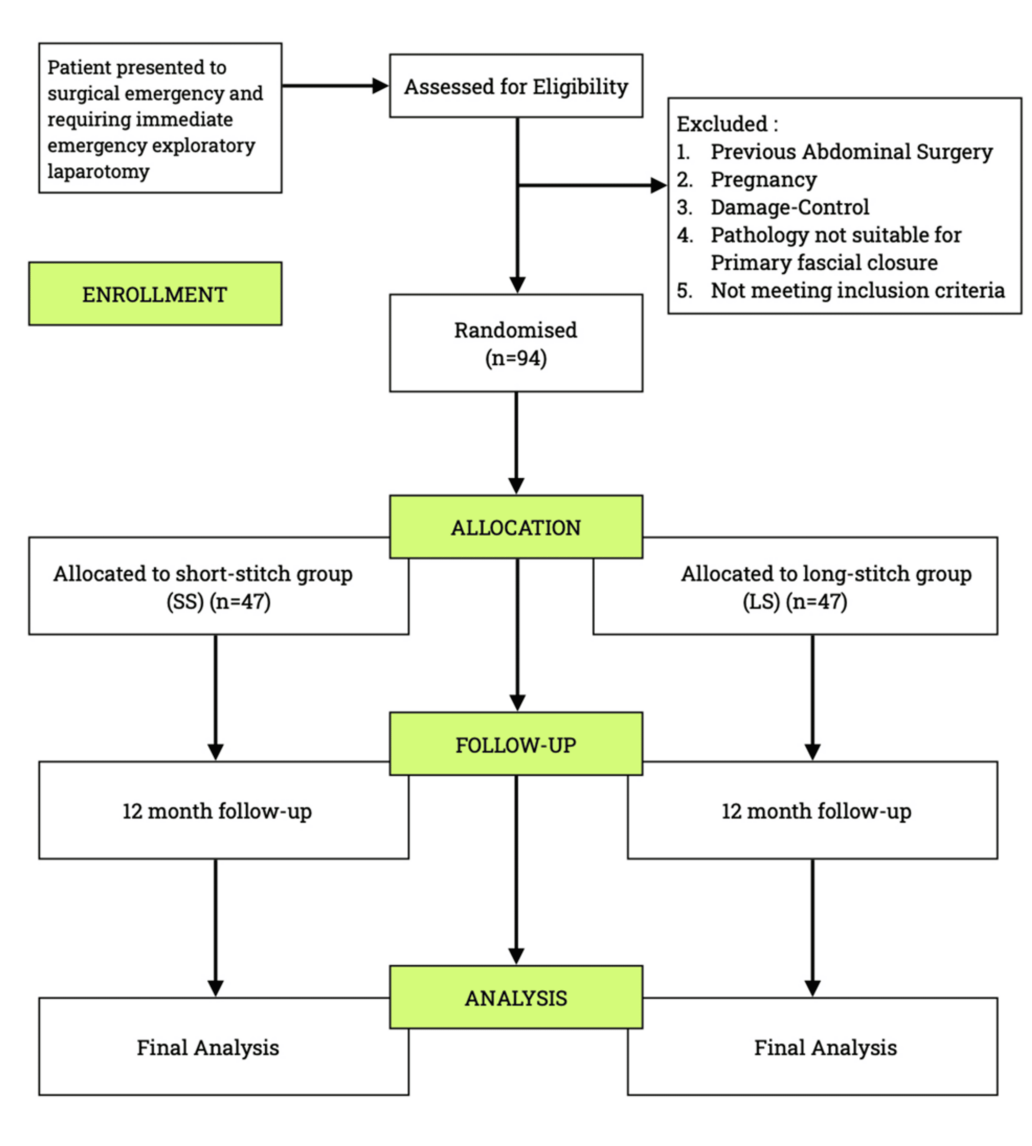
Flow diagram of the study

The study's flow is illustrated in Figure 1.

## Results

Demographics and clinical characteristics

The demographic and clinical characteristics were comparable between the LS and SS groups, with no statistically significant differences observed in age, gender distribution, anthropometric parameters, hemoglobin, or serum albumin levels (p > 0.05) when each of these parameters was compared individually to the incidence of SSI or SWD. The distribution among groups also showed no statistically significant differences (Table 1).

**Table 1:** Demographics and clinical characteristics (baseline values) t = t-value; calculated by applying an independent-samples t-test. χ² = chi-square value; calculated by applying Pearson's chi-square test. BMI: body mass index.

Parameter	Long-stitch group (n = 47)	Short-stitch group (n = 47)	Test statistic	p-value
Age (years)	35.8 ± 16.8	35.7 ± 15.2	t = 0.030	0.982
Female sex, n (%)	10 (21.3)	10 (21.3)	χ² = 0.000	1.000
Mean weight (kg)	48.4 ± 9.9	53.3 ± 11.3	t = 2.236	0.247
Mean height (cm)	145.7 ± 11.9	147.5 ± 11.8	t = 0.736	0.841
Mean BMI (kg/m²)	22.7 ± 4.0	24.9 ± 5.0	t = 2.355	0.458
Mean hemoglobin level (g/dL)	12.2 ± 2.0	12.5 ± 2.1	t = 0.709	0.612
Mean serum albumin level (g/dL)	3.3 ± 0.8	3.5 ± 1.6	t = 0.766	0.573

Comparison of the length of the midline incision and time-to-fascial closure

The mean incision length was comparable between the LS group (14.1 ± 2.8 cm) and the SS group (13.2 ± 3.7 cm), with no statistically significant difference (p = 0.247) (Table 2). However, the mean fascial closure time was significantly longer in the SS group (14.8 ± 4.2 minutes) compared to the LS group (11.4 ± 2.9 minutes) (p < 0.001). 

**Table 2:** Time-to-fascial closure and length of midline incision t = t-value; calculated by applying an independent-samples t-test.

Parameter	Long-stitch group (n = 47)	Short-stitch group (n = 47)	Test statistic	p-value
Mean incision length (cm)	14.1 ± 2.8	13.2 ± 3.7	t = 1.165	0.247
Mean time-to-fascial closure (min)	11.4 ± 2.9	14.8 ± 4.2	t = 4.567	<0.001

Comparison of SSI

Thirteen patients in the LS group and five patients in the SS group developed SSI during their index hospital stay. The incidence of SSI was higher in the LS group (27.7%) compared to the SS group (10.6%), with a statistically significant difference (p = 0.036) (Table 3). Although the proportion of severe SSI (Grade ≥ 4) was higher in the LS group, the difference was not statistically significant (p = 0.065). The incidence of SSI events across the different time-points, by postoperative day, is shown in Table 4.

**Table 3:** Incidence of surgical site infection (SSI) (from POD 1 until the end of POD 7) χ^2^ = chi-square value; calculated by applying Pearson's chi-square test for incidence of SSI among both groups. ^#^Overallincidence of SSI: new unique patients from the group developing an SSI event of any grade, from postoperative day 0 until the end of postoperative day 7. ^&^Severe SSI (Grade ≥ 4) incidence: new unique patients from the group developing an SSI event of Grade ≥ 4, from postoperative day 0 until the end of postoperative day 7. SSI: surgical site infection, POD: postoperative day.

Outcome	Long-stitch group (n = 47)	Short-stitch group (n = 47)	Test statistic	p-value
Overall SSI incidence^#^, n (%)	13 (27.7%)	5 (10.6%)	χ^2 ^=4.396	0.036
Severe SSI (Grade ≥ 4) incidence^&^, n (%)	7 (14.9%)	3 (6.4%)	χ^2 ^=3.405	0.065

**Table 4:** Incidence of SSI over time ^#^Incidence: new unique patients with an SSI event at the end of the given postoperative day. POD: postoperative day, SSI: surgical site infection.

Incidence^#^ of SSI over time	Long-stitch group (n = 47)	Short-stitch group (n = 47)
POD 1	2	1
POD 3	10	3
POD 5	1	1
POD 7	0	0

Comparison of SWD

The overall incidence of SWD was higher in the LS group (36.17%) compared to the SS group (12.77%), showing a statistically significant difference (p = 0.0164) (Table 5). Although severe SWDs (Grade ≥ 3) were more frequent in the LS group, the difference was not statistically significant (p = 0.094). The incidence of SWD events across the different time-points, by postoperative day, is shown in Table 6.

**Table 5:** Incidence of surgical wound dehiscence (SWD) (from POD 1 until the end of POD 7) χ^2^ = chi-square value; calculated by applying Pearson's chi-square test. ^#^Overall incidence of SWD: new unique patients from the group developing an SWD event of any grade, from postoperative day 0 until the end of postoperative day 7. ^&^Severe SWD (Grade ≥ 3) incidence: new unique patients from the group developing an SWD event of Grade ≥ 3, from postoperative day 0 until the end of postoperative day 7. POD: postoperative day, SWD: surgical wound dehiscence (graded according to the WUWHS-Sandy SWD Grading System [17]).

Parameter	Long-stitch group (n = 47)	Short-stitch group (n = 47)	Test statistic	p-value
Overall SWD incidence^#^, n (%)	17 (36.17%)	6 (12.77%)	χ^2^ = 5.7563	0.0164
Severe SWD (Grade ≥ 3)^&^, n (%)	8 (17.02%)	2 (4.26%)	χ^2^ = 2.7976	0.0944

**Table 6:** Incidence of surgical wound dehiscence over time ^#^Incidence: new unique patients with an SWD event at the end of the given postoperative day. POD: postoperative day.

Incidence^#^ of surgical wound dehiscence over time	Long-stitch group (n = 47)	Short-stitch group (n = 47)
POD 1	2	1
POD 3	10	3
POD 5	5	0
POD 7	0	2

**Figure 2:**
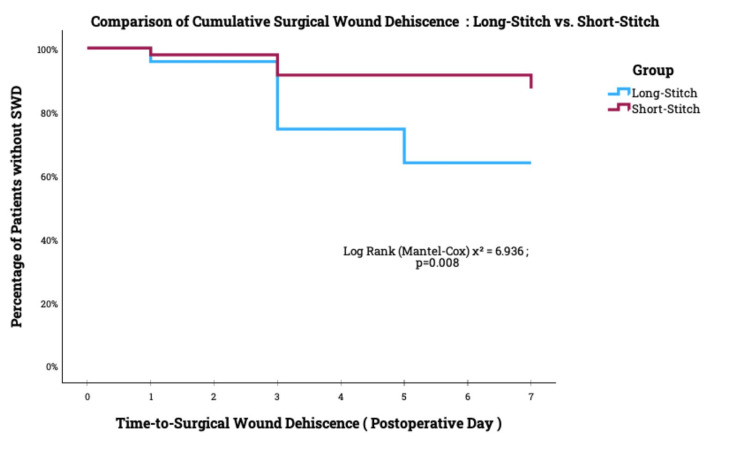
Kaplan-Meier curve: comparison of cumulative surgical wound dehiscence χ² = chi-square; calculated by the log-rank (Mantel-Cox) test. SWD: surgical wound dehiscence.

On performing a log-rank analysis to compare the time-to-SWD between the LS and SS groups, the mean time to SWD was significantly longer in the SS group (6.62 days; 95% CI: 6.22-7.02) compared to the LS group (5.68 days; 95% CI: 5.14-6.23). The difference was statistically significant by the log-rank (Mantel-Cox) test (χ^2 ^= 6.936, p = 0.008). The data are shown in Table 7. A Kaplan-Meier curve for this log-rank analysis was plotted and is shown in Figure 2.

**Table 7:** Mean time-to-surgical wound dehiscence (SWD) between groups: log-rank comparison χ^2 ^= chi-square, calculated by applying the log-rank (Mantel-Cox) test. ^#^Mean time is an estimate limited to the longest follow-up time for the event (i.e., postoperative day 7).

Group	Mean estimate^#^ (days)	95% CI	Test statistic	p-value
Long-stitch	5.68	5.14-6.23	-	
Short-stitch	6.62	6.22-7.02	-	
Overall	6.15	5.80-6.50	χ^2 ^= 6.936	0.008

Postoperative pain: comparison of postoperative VAS scores

Postoperative pain scores measured by VAS on PODs 1, 3, 5, and 7 were comparable between the LS and SS groups, with no statistically significant differences observed (p > 0.05). Similarly, the mean length of hospital stay was comparable between the LS group (11.2 ± 9.9 days) and the SS group (10.8 ± 8.5 days) (p = 0.574), with no statistically significant difference (Table 8). 

**Table 8:** Postoperative pain scores: stratified by VAS scores VAS: Visual Analogue Scale, POD: postoperative day. t = t-value; calculated by applying an independent-samples t-test.

Parameter	Long-stitch group (n = 47)	Short-stitch group (n = 47)	Test statistic	p-value
VAS POD 1	5.7 ± 1.4	5.6 ± 1.3	t = 0.447	0.656
VAS POD 3	4.1 ± 1.3	3.8 ± 1.5	t = 0.937	0.351
VAS POD 5	2.6 ± 1.2	2.2 ± 1.1	t = 1.437	0.154
VAS POD 7	1.4 ± 1.1	1.2 ± 0.7	t = 1.145	0.255

Comparison of the length of hospital stay

The mean length of hospital stay among participants in the LS group was 11.2±9.9 days, with a range of 3-41 days, while in the SS group, it was 10.8 ± 8.5 days, ranging from 3 to 35 days. Statistically, there was no significant difference between the two groups (p = 0.574) (Table 9).

**Table 9:** Length of hospital stay t = t-value; calculated by applying an independent-samples t-test.

Parameter	Long-stitch group (n = 47)	Short-stitch group (n = 47)	Test statistic	p-value
Mean length of hospital stay in days	11.2 ±9.9	10.8 ±8.5	t = 0.564	0.574

Comparison of the incidence of incisional hernia

At the 12-month mark, the incidence of incisional hernia by clinical examination was 29.79% (n = 14/47) in the LS group versus 10.64% (n = 5/47) in the SS group. The difference was statistically significant (p = 0.038) (Table 10). The calculated relative risk (RR) was 2.80. Thus, patients closed with the LS technique were 2.8 times more likely to develop an incisional hernia in the long term compared to those closed with the SS technique.

**Table 10:** Incidence of incisional hernia χ^2^ = chi-square value; calculated by applying Pearson's chi-square test.

Parameter	Long-stitch group (n = 47)	Short-stitch group (n = 47)	Test statistic	p-value
Incidence of incisional hernia at 12 months, n (%)	14 (29.79%)	5 (10.64%)	χ^2^ = 4.221	0.038

## Discussion

This prospective comparative study demonstrates that SS fascial closure following emergency exploratory laparotomy significantly reduces the incidence of SSI, SWD, and 12-month incisional hernia rates compared to the conventional LS technique and is a viable and safe alternative. These findings are clinically important, as wound-related morbidity remains substantially higher after emergency laparotomy than elective abdominal surgery [1].

The reduced incidence of SSI observed with short-stitch closure can be explained by improved tissue perfusion and reduced ischemia at the wound edges. Experimental studies have shown that smaller tissue bites preserve microcirculation and minimize tissue strangulation, thereby lowering the risk of infection [5]. Similar benefits of SS or small-bite techniques have been demonstrated in landmark randomized trials and meta-analyses conducted in elective laparotomy [3].

SWD was also less frequent in the SS group. Biomechanical evidence suggests that smaller tissue bites allow more uniform distribution of tension along the incision, providing greater resistance to fascial separation and wound failure [14]. This advantage is particularly relevant in emergency laparotomy patients, who are prone to increased intra-abdominal pressure in the postoperative period [2]. And over a longer time period, this leads to a decreased rate of incisional hernia. This is reflected in the statistically significant delay/prevention of dehiscence as well as reduced incidence of incisional hernia, shown in our analysis. 

Although SS closure required a modestly longer closure time, this increase was clinically acceptable and did not adversely affect postoperative pain or length of hospital stay. Comparable observations regarding closure time and postoperative outcomes have been reported in previous clinical trials [8].

SS closure is a current standard of care in elective laparotomy following the results of the STITCH trial [3] and the ESTOIH Trial [7]; however, there is little concrete evidence of its benefit in emergency settings. We are also aware of the CONTINT Trial [18], which compared interrupted versus continuous closure techniques in an emergency setting and found both techniques comparable. 

Limitations of the current study include a relatively small sample size. The follow-up duration was also relatively short, and even though early incisional hernias were successfully captured, a longer follow-up would have been ideal. Additionally, we have refrained from taking into account any reoperation or the length of negative-pressure vacuum therapy required for the management of the primary wound, or other such adjuncts, in our analysis.

## Conclusions

SS fascial closure following emergency exploratory laparotomy significantly reduces SSI and SWD compared to LS closure, with no increase in postoperative pain or hospital stay. It also offers meaningful benefits in long-term outcomes by reducing incisional hernia rates. And despite a modest increase in closure time, the technique offers meaningful clinical benefits and should be considered for routine use in emergency abdominal surgery.
